# Surgical Outcomes of XEN45 Gel Stent Using Ab Interno Technique in Open-Angle Glaucoma: A 2-Year Follow-Up Study

**DOI:** 10.3390/jcm14134617

**Published:** 2025-06-30

**Authors:** Doah Kim, Myungjin Kim, Marvin Lee, Seungsoo Rho

**Affiliations:** 1 Department of Ophthalmology, CHA Bundang Medical Center, CHA University, Seongnam 13496, Gyeonggi-do, Republic of Korea; inhamed93216006@gmail.com (D.K.); brandmjeyes@gmail.com (M.K.); 2 Allbarun Eye Clinic, Suwon 16499, Gyeonggi-do, Republic of Korea; mvdevil@naver.com

**Keywords:** ab interno, bleb, intraocular pressure, MIGS, minimally invasive glaucoma surgery, open-angle glaucoma, XEN gel stent

## Abstract

**Background/Objectives**: This study aims to evaluate the long-term efficacy and safety of ab interno techniques using minimally invasive glaucoma surgery (MIGS), specifically XEN gel stent implantation, by evaluating its 2-year outcomes in patients with primary open-angle glaucoma (POAG) and pseudoexfoliation glaucoma (PXG). **Methods**: This retrospective single-center study consecutively included 31 eyes of 31 patients with POAG or PXG who underwent XEN gel stent implantation. Patients were followed for 24 months, with assessments at multiple time points. Success was defined as achieving an IOP of less than 14 mmHg and a reduction of more than 20% from preoperative IOP without additional glaucoma surgery. Bleb morphology was evaluated using anterior segment optical coherence tomography (AS-OCT) and slit-lamp photographs. Postoperative interventions and complications were also recorded. **Results**: At 24 months, complete success and qualified success rates were 35.5% (11/31) and 51.6% (16/31), respectively. There was no difference in surgical success rates at 2 years based on the tip location (intraconjunctiva, intratenon, and uviform) on the 1st postoperative day. Patients with high sparse wall on AS-OCT imaging or avascular bleb morphology via slit-lamp photography at 6 months postoperatively had higher complete success rates at 2 years than those without (*p* = 0.007, *p* = 0.009, respectively). Patients with avascular bleb types at 6 months postoperatively had higher qualified success rates at 2 years compared with the vascular types (*p* = 0.038). Needling was performed in 32.3% of eyes, with secondary surgical procedures required in 16.1% of eyes. The most common adverse event was hypotony, occurring in 67.7% of eyes on the 1st postoperative day but resolving within 6 months. **Conclusions**: The ab interno XEN gel stent is an effective and minimally invasive option for managing POAG and PXG, with long-term success predicted by the AS-OCT assessment of bleb morphology at 6 months. Proactive postoperative management, emphasizing early intervention and monitoring, is crucial for maintaining optimal outcomes.

## 1. Introduction

Ab interno techniques in minimally invasive glaucoma surgery (MIGS) have transformed the landscape of glaucoma management by offering less invasive alternatives to traditional filtration surgeries like trabeculectomy and glaucoma drainage devices (GDDs) for patients with early to moderate glaucoma [1,2]. These traditional surgeries are associated with high complication rates, prompting the widespread adoption of MIGS procedures in recent years, particularly in the United States from 2013 to 2018 [3].

Among the array of MIGS procedures available, the XEN gel stent (Allergan, Dublin, CA, USA) has emerged as a notable option. Designed for ab interno placement, the XEN gel stent acts as a subconjunctival drainage device, effectively lowering intraocular pressure (IOP) while demonstrating safety and efficacy profiles comparable to trabeculectomy [4,5]. The ab interno approach minimizes surgical trauma, reduces postoperative complications such as hyphema, and preserves corneal endothelial integrity [6,7,8]. By sparing the conjunctiva from extensive manipulation, this technique aims to prevent subconjunctival bleeding and enhance the physiological absorption of aqueous humor through preserved conjunctival lymphatics.

Studies have shown that the XEN gel stent effectively reduces IOP and medication burden in patients with primary open-angle glaucoma (POAG) and pseudoexfoliation glaucoma (PXG) [9,10,11]. Comparable outcomes have been reported for both standalone XEN procedures and those combined with cataract surgery in several studies [12,13,14]. Compared with traditional filtering surgeries such as trabeculectomy, the XEN gel stent offers a favorable safety profile with a lower incidence of complications [15,16]. In addition, bleb morphology evaluated by anterior segment OCT has been proposed as a meaningful predictor of long-term surgical success [17,18].

In our previous study, we conducted a 6-month follow-up on patients who underwent XEN gel stent implantation and identified key predictors of surgical success by evaluating clinical parameters and bleb morphology using anterior segment optical coherence tomography (AS-OCT) and slit-lamp photographs [17]. The short-term outcomes demonstrated promising reductions in IOP and medication dependency, validating the efficacy and safety of the XEN gel stent in a conjunctiva-sparing approach.

Based on previous research findings, this study extended the follow-up period to 24 months for the same patient cohort to examine the long-term efficacy, safety, and stability of the XEN gel stent. By analyzing the evolution of bleb morphology and clinical outcomes using AS-OCT images and slit-lamp examinations, our goal is to identify reliable predictors of sustained surgical success. This research aims to significantly advance the understanding of MIGS, particularly the ab interno approach with the XEN gel stent, providing crucial insights for optimizing glaucoma management strategies.

## 2. Materials and Methods

### 2.1. Study Enrollment

In this retrospective single-center study, the electronic medical records of all patients who underwent XEN gel stent surgery for medically uncontrolled POAG or PXG at the CHA Bundang Medical Center between November 2018 and June 2019 were reviewed and consecutively enrolled. This study was approved by the Institutional Review Board of the CHA Bundang Medical Center and conducted at the CHA Glaucoma Clinic of the CHA Bundang Medical Center in accordance with the tenets of the Declaration of Helsinki. Written informed consent was obtained from all patients without a waiver.

### 2.2. Surgical Success Definitions

Success was defined as achieving an IOP of less than 14 mmHg and more than 20% reduction in preoperative IOP, with no additional glaucoma surgery or findings of vision-threatening complications. This includes qualified success, with or without any ocular hypotensive medications, and complete success, without any ocular hypotensive medications. Needling was not considered as glaucoma surgery. Hypotony was defined as having an IOP of less than 6 mm Hg at any visit.

### 2.3. Surgical Procedures

All surgical procedures were performed by a skilled surgeon (S.R.) after obtaining informed consent from all patients, following procedures outlined in previous studies [17]. Briefly, after topical anesthesia, 0.05 mL of 2% lidocaine with epinephrine was injected into the superior subconjunctival space, 6 mm from the XEN tip site. Viscoelastics were injected via a 1 mm side port to maintain the anterior chamber. The XEN injector, through a 1.5 mm corneal incision, placed the XEN implant 2 mm from the limbus at the superonasal angle. Confirmation via surgical gonioscopy was followed by viscoelastic removal, corneal wound sealing with balanced salt solution hydrosealing, and subconjunctival MMC injection (0.05 mL, 0.2–0.4 mg/mL).

### 2.4. Follow-Up and Outcome Measures

Patients were followed up on postoperative day 1, week 1, month 1, month 3, month 6, month 12, month 18, and month 24. During each visit, IOP measurement, best-corrected visual acuity (BCVA) assessment, slit-lamp examination, corneal endothelial cell count examination, and AS-OCT were performed. The primary outcome evaluated the surgical success rate by assessing the reduction in IOP and the change in the number of medications compared with baseline in the 2-year follow-up after XEN gel stent surgery. Secondary outcome measures included evaluating long-term surgical outcomes at 2 years based on bleb morphology classified early postoperatively by AS-OCT or slit-lamp examination after surgery. The tip location of the XEN stent was categorized into three groups based on its anatomical position: intraconjunctival, when the tip was located beneath the conjunctiva; intratenon, when the tip was positioned along the interface between the conjunctiva and Tenon’s capsule; and uviform, when the tip was obscured by multiple protuberances within a poorly hydrated bleb, making precise localization difficult.

### 2.5. Statistical Analysis

All data were analyzed using SPSS software version 29.0 (IBM Corp., Armonk, NY, USA). Descriptive statistics were used to summarize the baseline characteristics of the study population. Continuous variables were represented as mean ± standard deviation (SD), while categorical variables were represented as frequencies and percentages. All statistical tests were two-tailed, and a *p*-value < 0.05 was considered statistically significant. The baseline characteristics were compared between the success and failure groups. Independent t-tests were used for continuous variables such as age, axial length, central corneal thickness (CCT), preoperative visual field index (VFI), preoperative IOP, the number of preoperative medications, and postoperative 1 week IOP. For categorical variables such as sex, the presence of POAG, and PXG, Fisher’s exact test was employed to assess the association between these variables and surgical outcomes. The Kruskal–Wallis test was conducted to compare the means among three independent groups due to the small sample size in each group. To evaluate the postoperative course of IOP, number of medications, and ECC changes over time, repeated measures ANOVA was employed to compare the mean values at multiple time points (preoperative, 1 month, 6 months, 12 months, 18 months, and 24 months) within each group (complete success and qualified success). A post hoc test was conducted to identify specific time points where significant differences occurred between the groups. For the non-parametric data related to the number of medications, the Wilcoxon signed-rank test was used to calculate statistical significance. For the analysis of success or failure at 2 years based on bleb morphology via AS-OCT imaging and slit-lamp photography, Fisher’s exact test was used due to the relatively small sample sizes in some of the bleb morphology categories.

## 3. Results

### 3.1. Demographics of Success and Failure Groups

A standard ab interno XEN implantation was performed in 31 eyes of 31 patients who were followed for at least 2 years. According to the definition of success, as described in the Methods section, the demographic features of the groups that achieved complete success versus failure and qualified success versus failure are summarized in Table 1. No statistically significant difference was observed between the complete success and failure groups, as well as the qualified success and failure groups, regarding age, sex, axial length, CCT, preoperative VFI, preoperative IOP, preoperative medications, the concentration of MMC (0.02% or 0.04%) used, and proportion of POAG and PXG.

### 3.2. Surgical Success

**Figure 1:**
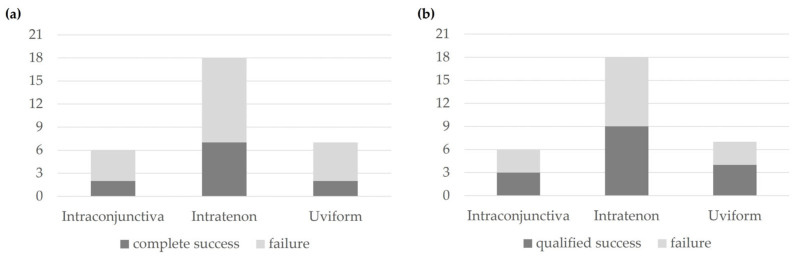
Success or failure at the 2 years of complete success (**a**) and qualified success (**b**) rates by the number of patients in relation to the intraconjunctiva, intratenon, and uviform tip locations based on the tip location on the 1st postoperative day. (**a**) *p* = 0.268, (**b**) *p* = 0.637 (Kruskal–Wallis test).

Based on the tip location on postoperative day 1, the success rates at 2 years postoperatively were assessed. For complete success, the rates were 33.3% for intraconjunctiva, 38.9% for intratenon, and 28.6% for uviform tip locations (Figure 1a). For qualified success, the rates were 50.0% for intraconjunctiva, 50.0% for intratenon, and 57.1% for uviform tip locations (Figure 1b). The Kruskal–Wallis test indicated that the differences in success rates among the intraconjunctiva, intratenon, and uviform groups were not statistically significant for both complete success and qualified success (*p* = 0.268 and *p* = 0.637, respectively).

**Figure 2:**
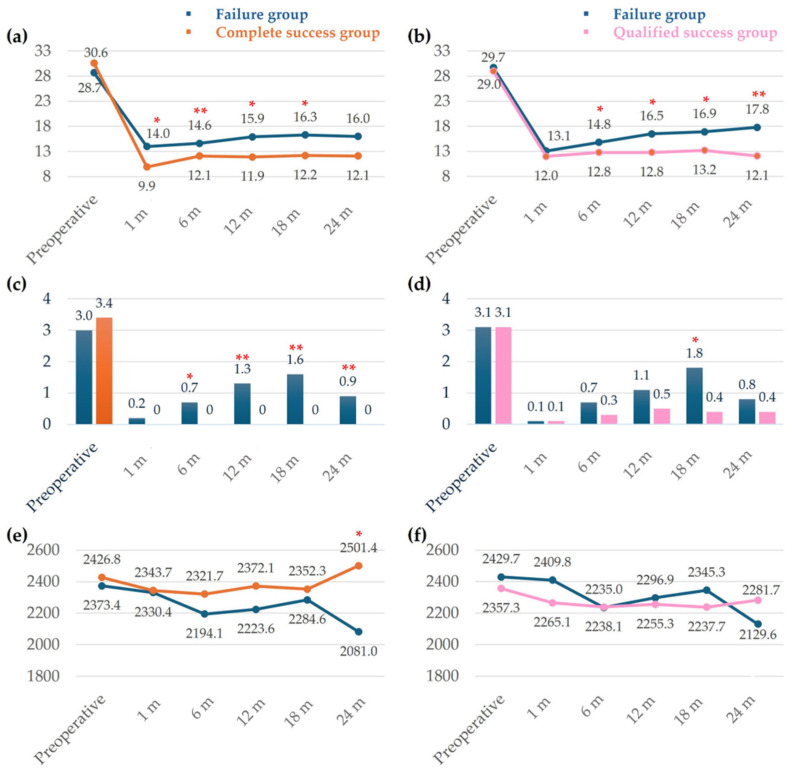
Mean IOP changes (**a**,**b**), number of medications (**c**,**d**), and change in ECC (**e**,**f**) over time in the complete success group (**a**,**c**,**e**) and qualified success group (**b**,**d**,**f**) compared with the failure group preoperatively and at 1 month, 6 months, 12 months, 18 months, and 24 months postoperatively. IOP, intraocular pressure (mmHg); ECC, endothelial cell count (*, *p* < 0.05; **, *p* < 0.01).

In the complete success group, IOP was significantly lower at 1, 6, 12, 18, and 24 months postoperatively compared with the failure group (Figure 2a). In the qualified success group, IOP remained consistently lower than in the failure group after 6 months postoperatively (Figure 2b). The number of intraocular pressure-lowering medications was not significantly different between the success groups (qualified and complete success) and the two failure groups preoperatively. However, the complete success group required consistently fewer medications than the failure group after 6 months postoperatively (Figure 2c). The qualified success group did not show a statistically significant difference in the number of medications at any time point except at 18 months postoperatively (Figure 2d). The change in endothelial cell count (ECC) showed a significant difference between the complete success and failure groups at 2 years, but no statistically significant differences were observed between the success groups (qualified and complete success) and the two failure groups at other times.

**Figure 3:**
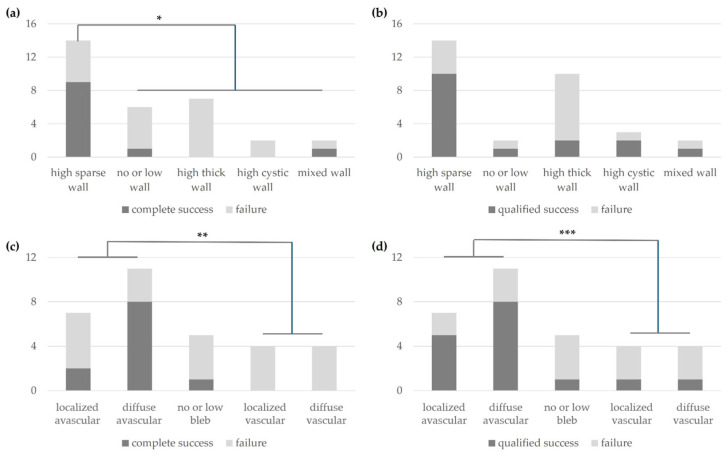
Success or failure at the 2 years of complete success (**a**) or qualified success (**b**) according to bleb morphology via AS-OCT imaging and complete success (**c**) or qualified success (**d**) according to bleb morphology via slit-lamp photography at 6 months postoperatively (*, *p* = 0.007; **, *p* = 0.009; ***, *p* = 0.038).

Based on the analysis of bleb morphology using AS-OCT at 6 months postoperatively, the surgical success at 2 years postoperatively was evaluated. In the complete success group, the high sparse wall group at 6 months postoperatively had a statistically significantly higher success rate compared with other morphologies (*p* = 0.007) (Figure 3a). In the qualified success group, there was no statistically significant difference in success rate at 2 years based on bleb morphology (Figure 3b). Based on the classification of bleb morphology groups using slit-lamp photography at 6 months postoperatively, the surgical success rate at 2 years was significantly higher in the avascular group than in the vascular group for both complete success (*p* = 0.009) (Figure 3c) and qualified success groups (*p* = 0.038) (Figure 3d).

**Figure 4:**
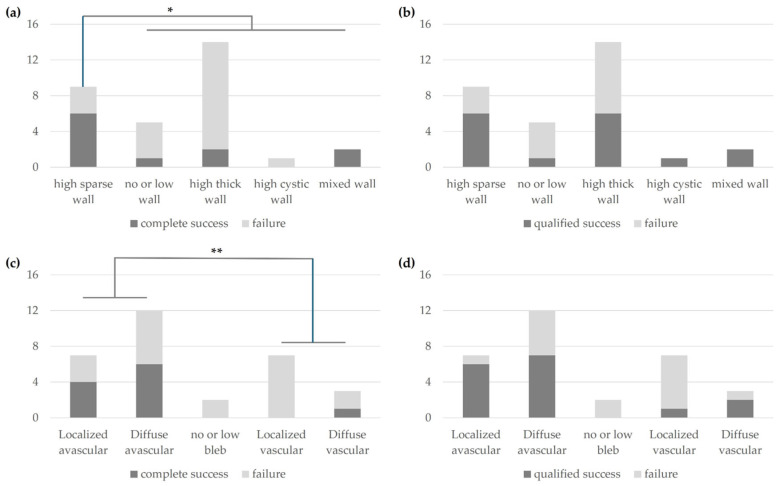
Success or failure at 2 years of complete success (**a**) or qualified success (**b**) according to bleb morphology via AS-OCT imaging and complete success (**c**) or qualified success (**d**) according to bleb morphology via slit-lamp photography at 2 years postoperatively (*, *p* = 0.038; **, *p* = 0.044).

At 2 years postoperatively, complete success was most observed in the high sparse wall type (*p* = 0.038) as classified by AS-OCT (Figure 4a), while there was no difference in success rates among bleb morphologies for qualified success (Figure 4b). Additionally, based on slit-lamp photography at 2 years postoperatively, the avascular type demonstrated a higher surgical success rate compared with the vascular type (*p* = 0.044) (Figure 4c), with no difference observed based on bleb morphology for qualified success (Figure 4d).

### 3.3. Postoperative Interventions

Needling was performed in 10 eyes (32.3%) out of 31, and a total of 18 needling procedures were performed. Among the eyes that received needling, the proportion of those requiring one and two procedures was the same (12.9%), while 2 eyes (6.5%) underwent three needling procedures. A secondary surgical procedure was performed in a total of 5 eyes (16.1%), with 3 eyes undergoing trabeculectomy and 3 eyes (9.7%) and 2 eyes (6.5%) undergoing XEN revision (Table 2).

### 3.4. Safety

Regarding the safety profile, hypotony was the most common complication. It occurred in 21 eyes (67.7%) on the 1st postoperative day, 7 eyes (22.6%) at 1 week postoperatively, and 1 eye (3.2%) at 1 month postoperatively. There were no cases of hypotony at 6 months, 1 year, or 2 years postoperatively. The incidence of ocular adverse events, excluding hypotony, was observed in 10 eyes (32.3%). Hyphema was the most common, occurring in 4 eyes (12.9%) and resolved spontaneously within 14 days postoperatively. Choroidal detachment occurred in 3 eyes (9.5%). Vitreous hemorrhage and tube bending each occurred in 2 eyes (6.5%), while tube migration occurred in 1 eye (3.2%) (Table 3).

## 4. Discussion

One of the most notable observations in this study is the association between bleb morphology observed through slit-lamp examination and AS-OCT imaging at 6 months postoperatively and surgical success at 2 years. While this finding suggests a potential predictive value, further validation in larger prospective studies is warranted. Notably, patients with high sparse wall or avascular bleb morphology at 6 months tended to maintain higher surgical success rates at 2 years. Using AS-OCT, known for its high reliability in evaluating intraocular structures and providing accurate repeated measurements [19], Lenzhofer et al. reported that the XEN gel stent resulted in a higher proportion of uniform blebs (48%) compared with trabeculectomy, with significantly greater bleb height and internal cavity presence at 6 months (*p* = 0.031, *p* < 0.001) and 1 year (*p* = 0.039, *p* = 0.001), and lower postoperative IOP in the internal cavity group at both time points (*p* = 0.024, *p* = 0.040) [20]. These findings align with Seoyoung Wy’s observations that blebs with an internal cavity, or diffuse subconjunctival cysts, had lower postoperative IOP and better regulated aqueous humor outflow due to the cavity height [18]. These bleb morphologies suggest that AS-OCT imaging can serve as an important indicator for predicting long-term surgical success, along with more stable IOP reduction. Our previous study on the 6-month follow-up data revealed that postoperative IOP at 1 week and female gender were significantly associated with higher success rates [17]. This suggested that early postoperative IOP and gender could be potential predictors of surgical success in the short term. However, in the current study’s long-term follow-up at 2 years, we observed that neither postoperative IOP at 1 week nor gender had a significant impact on the success rates. This discrepancy between the short-term and long-term predictors highlights the dynamic nature of surgical outcomes and suggests that early indicators might not necessarily translate into long-term success. This discrepancy may be due to the progressive remodeling of the bleb and surrounding tissues over time, which cannot be fully predicted by early IOP alone. Initial IOP reduction may reflect immediate surgical success but not necessarily indicate sustained outflow function. These findings underscore the importance of mid-term assessments, such as AS-OCT imaging at 6 months, as more reliable indicators of long-term outcomes, and highlight the need for continued postoperative monitoring beyond the early postoperative period. They also emphasize the importance of evaluating multiple factors over an extended period to accurately predict surgical outcomes. Additionally, it is important to note that bleb morphology can change over time with follow-up, potentially affecting long-term success rates. This shift in predictive factors from short-term to long-term outcomes is an intriguing aspect of our study, shedding light on the complex and evolving nature of bleb morphology and its influence on surgical success.

In a large-scale retrospective multicenter observational study involving 646 eyes, the complete success (6 ≤ IOP ≤ 18 mmHg, no medication) and qualified success (6 ≤ IOP ≤ 18 mmHg, with medication) rates at 2 years postoperatively were 26% and 48%, respectively [21]. Reitsamer et al. reported a clinical success rate (≥20% IOP reduction, same or fewer medications without secondary surgical intervention) of 67.6% at 12 months and 65.8% at 24 months [22]. Grover et al. evaluated the 12-month outcomes of standalone XEN implantation in 65 patients with refractory glaucoma, finding that 75.4% of eyes achieved more than a 20% reduction in IOP while using a similar or fewer number of medications [23]. In a cohort of 129 eyes, 54.2% achieved more than a 20% reduction in IOP and maintained an IOP of less than 18 mmHg for 24 months following standalone XEN surgery [24]. Using the 16 mmHg threshold, 51.4% (POAG) versus 57.1% (PXG) eyes achieved complete success (*p* = 0.70) at 2 years [25]. This shows that the notable advantage of the PXG group over the POAG cohort during the first 12 months is no longer statistically significant, as observed by the same authors. We defined the success criterion for IOP as being less than 14 mmHg, which is even more strict than in other study designs. In our study, the complete success rate at 2 years was 35.5% (11/31), and the qualified success rate was 51.6% (16/31), which seems to have lower success rates compared with other studies. Rauchegger et al. reported that complete surgical success was achieved in 39% of patients at 12 months and 34% at 24 months, with qualified success in 29% at 12 months and 27% at 24 months, while 13 eyes (16%) were classified as complete surgical failure [9]. The minimal change in success rates from 12 months to 24 months postoperatively highlights the long-term stability of the XEN gel stent. Thus, the XEN gel stent can be considered a safe and effective long-term treatment option for glaucoma.

In our study, needling was performed in 10 out of 31 eyes (32.3%), with a total of 18 needling procedures conducted. Patrica et al. reported a needling rate of 19% in 94 patients who underwent a standardized needling technique over 1 year [26], while Grover et al. evaluated 65 patients with refractory glaucoma who received XEN45 with mitomycin C 0.2 mg/mL and disclosed needling rates of 32% [23]. A study performed in Portugal involving 15 eyes undergoing XEN with MMC 0.2 mg/mL had a 33% needling rate 3 months after surgery [26]. A recent study published by Midha et al. reported an overall needling rate of 45% over 24 months [27]. Reitsamer et al. and Gabbay et al. reported needling rates of 41.1% and 37.7%, respectively, after 2 years of follow-up [22,28], while Tan et al. reported a needling rate of 51.3% after 1 year [10]. High needling rate could be attributed to our proactive approach to managing bleb function early in the postoperative period. Ensuring optimal bleb function is crucial in the early months following surgery to prevent fibrosis and scarring, which can lead to surgical failure.

The use of intraoperative MMC during XEN implant surgery has become standard practice [1,2,5,7,9,17,18,20,21,22,23,26]. Natalia et al. investigated the efficacy and safety of two MMC doses (0.01% vs. 0.02%) in eyes undergoing XEN45 implantation, either alone or with phacoemulsification, and found no significant differences in IOP reduction, hypotensive medication use, or adverse events between the doses [29]. The results suggest that lower doses might be feasible, although MMC 0.01% did not reduce adverse events [29]. A study on standalone XEN45 gel stent implantation reported a therapeutic success rate of 39% without MMC and 55% with MMC at 1 year, while the failure rates were 61% without MMC and 45% with MMC, indicating that MMC use appeared to increase the therapeutic success rate, though it did not reach statistical significance [30].

Regarding XEN45, the concentration of MMC could potentially affect clinical outcomes. Some research suggests that the success rate might be linked to the MMC dose, although other studies have found no correlation between MMC dosage and surgical results [31]. In our study, we used MMC at concentrations of 0.02% and 0.04%, but no significant difference in success rates was observed between these concentrations, suggesting that the concentration of MMC may not be a critical factor in determining the success of the procedure. Additionally, there is a report of severe complication where MMC toxicity led to significant eye pain and a large avascular bleb [32].

Currently, there is no definitive evidence to support the use of a particular MMC concentration. Therefore, it might be advisable to use the lowest effective dose of MMC as judged by the surgeon for the specific patient. Nonetheless, further research is needed to provide clearer guidance on this matter. In particular, identifying the optimal and least hazardous concentration of MMC should be a key focus of future investigations.

XEN has emerged as a safe and less invasive method for lowering IOP in glaucoma patients, but it is still not free from complications. Hypotony (IOP < 6 mmHg) is the most common complication, occurring in approximately 4.0–27.0% of cases [21,22,33,34]. Nicolaou et al. reported that numerical hypotony (IOP  ≤ 6 mmHg) occurred at any point in 75 of 186 cases (40%), and no significant visual acuity deficit remained 4 weeks after surgery [12]. In our study, we observed a hypotony rate of 67.6% (10 eyes) on the 1st postoperative day. The higher rate of hypotony in our study compared with that in other studies may be attributed to the fact that we included measurements taken on the 1st postoperative day, whereas most other studies measured the incidence at 1 month or 6 months postoperatively. However, the hypotony rate in our study decreased to 3.2% (1 eye) by the 1st month postoperatively and was not observed again. Notably, all cases of hypotony resolved within 2 to 3 months without long-term adverse effects, highlighting the transient nature of this complication when managed properly.

Secondary glaucoma surgeries following XEN implantation have been reported to occur in 10.4–41.5% of cases [9,21,22,34,35,36]. Our study observed that secondary surgical procedures were necessary in 5 out of 31 eyes (16.1%) within the 1st 2 years, with 3 eyes undergoing trabeculectomy and 2 eyes undergoing XEN revision. This finding aligns with that of Rauchegger et al., who reported a 16% (*n* = 13) rate of secondary interventions, including trabeculectomy (11%, *n* = 9) and repeat XEN implantation (5%, *n* = 4) [9]. This indicates that, despite the initial success of the XEN gel stent, some patients still required additional surgical intervention to maintain adequate IOP control regarding stent occlusion or bleb fibrosis.

This study, despite its small sample size, followed up all 31 patients from a previously reported study for a period of 2 years after surgery, providing long-term outcome data. This comprehensive follow-up underscores that our findings reflect real-world data. Additionally, this study focused on naïve patients with no prior glaucoma surgery, which limits direct comparisons with the outcomes of XEN implantation in patients with secondary glaucoma. Lastly, the criteria for needling were based on AS-OCT morphology and the absence of tube occlusion, with YAG laser being used for cases of tube occlusion. Therefore, the needling procedures and their timing were not standardized and were left to the discretion of the experienced surgeon (S.R.). This variability could influence the outcomes and makes it challenging to draw definitive conclusions about the efficacy of needling in managing bleb function. Standardized protocols for needling interventions should be established and evaluated in future studies to determine the optimal timing and technique for improving surgical outcomes. In addition, comparative studies with other MIGS, such as iStent, Hydrus, or Preserflo, would provide further insights on the relative efficacy and safety of the XEN gel stent and help clarify its clinical role among current surgical options. Furthermore, long-term follow-up beyond 2 years is warranted to evaluate the sustained effectiveness and safety of the XEN gel stent and to determine whether the favorable outcomes observed in this study are maintained over time.

## 5. Conclusions

In conclusion, the ab interno XEN gel stent showed favorable 2-year outcomes in patients with POAG and PXG. High sparse wall and avascular bleb types at 6 months were associated with greater long-term success, highlighting the prognostic value of mid-term AS-OCT imaging. These findings support the importance of structured postoperative monitoring in sustaining surgical efficacy.

## Figures and Tables

**Table 1:** Clinical characteristics of patients treated with XEN gel stent.

	Qualified Success			Complete Success		
	No (*n* = 15)	Yes (*n* = 16)	*p*-Value	No (*n* = 20)	Yes (*n* = 11)	*p*-Value
Age	64.20 ± 15.51	69.13 ± 15.03	0.874	66.40 ± 14.57	67.36 ± 17.04	0.408
Sex (female, %)	8 (57.1%)	6 (42.9%)	1.000	6 (46.2%)	7 (53.8%)	0.449
Axial length (mm)	25.39	24.69	0.260	25.06	24.98	0.820
CCT (um)	513.33	528.44	0.066	519.10	524.	0.322
Preoperative VFI (%)	44.33	55.13	0.329	45.45	58.3	0.291
Preoperative IOP (mmHg)	29.67	29.0	0.905	28.65	30.6	0.576
Preoperative medications (*n*)	3.13 ± 1.13	3.06 ± 1.24	0.965	2.95 ± 1.23	3.36 ± 1.03	0.576
MMC			0.504			0.273
0.02% MMC	60.0%	43.7%		60.0%	36.4%	
0.04% MMC	40.0%	56.3%		40.0%	63.6%	
POAG (%)	53.3	81.3	0.135	55.0	90.9	0.055
PXG (%)	46.7	18.7	0.135	45.0	9.1	0.055

sCCT, central corneal thickness; VFI, visual field index; IOP, intraocular pressure; MMC, mitomycin C; POAG, primary open-angle glaucoma; PXG, pseudoexfoliative glaucoma. Values are presented as mean ± standard deviation unless otherwise indicated.

**Table 2:** Postoperative interventions and secondary surgical procedure.

Interventions	Number of Eyes (%) (*n* = 31)
Needling	10 (32.3)
1×	4 (12.9)
2×	4 (12.9)
3×	2 (6.5)
Secondary surgical procedure	5 (16.1)
Trabeculectomy	3 (9.7)
XEN revision	2 (6.5)

**Table 3:** Adverse events reported throughout the 2-year follow-up. * Intraocular pressure < 6 mmHg.

Adverse Events	Number of Eyes (%) (*n* = 31)
Hypotony *	
POD 1d	21 (67.7)
POD 1w	7 (22.6)
POD 1m	1 (3.2)
POD 6m	0 (0.0)
POD 12m	0 (0.0)
POD 24m	0 (0.0)
Hyphema	4 (12.9)
Vitreous hemorrhage	2 (6.5)
Tube bending	2 (6.5)
Tube migration	1 (3.2)
Choroidal detachment	1 (3.2)

## Data Availability

The datasets analyzed during the current study are available from the corresponding author on reasonable requests.
